# Nutritional management in head and neck cancer: United Kingdom National Multidisciplinary Guidelines

**DOI:** 10.1017/S0022215116000402

**Published:** 2016-05

**Authors:** B Talwar, R Donnelly, R Skelly, M Donaldson

**Affiliations:** 1 Head and Neck Centre, University College London Hospital NHS Foundation Trust, London, UK; 2 Department of Nutrition and Dietetics, Guy's and St Thomas’ NHS Foundation Trust, London, UK; 3 Department of Nutrition and Dietetics, Aintree University Hospitals NHS Foundation Trust, Liverpool, UK; 4 Department of Dietetics and Nutrition, QMC Campus, Nottingham University Hospitals NHS Trust, Nottingham, UK

## Abstract

Nutritional support and intervention is an integral component of head and neck cancer management. Patients can be malnourished at presentation, and the majority of patients undergoing treatment for head and neck cancer will need nutritional support. This paper summarises aspects of nutritional considerations for this patient group and provides recommendations for the practising clinician.

**Recommendations:**

• A specialist dietitian should be part of the multidisciplinary team for treating head and neck cancer patients throughout the continuum of care as frequent dietetic contact has been shown to have enhanced outcomes. (R)

• Patients with head and neck cancer should be nutritionally screened using a validated screening tool at diagnosis and then repeated at intervals through each stage of treatment. (R)

• Patients at high risk should be referred to the dietitian for early intervention. (R)

• Offer treatment for malnutrition and appropriate nutrition support without delay given the adverse impact on clinical, patient reported and financial outcomes. (R)

• Use a validated nutrition assessment tool (e.g. scored Patient Generated–Subjective Global Assessment or Subjective Global Assessment) to assess nutritional status. (R)

• Offer pre-treatment assessment prior to any treatment as intervention aims to improve, maintain or reduce decline in nutritional status of head and neck cancer patients who have malnutrition or are at risk of malnutrition. (G)

• Patients identified as well-nourished at baseline but whose treatment may impact on their future nutritional status should receive dietetic assessment and intervention at any stage of the pathway. (G)

• Aim for energy intakes of at least 30 kcal/kg/day. As energy requirements may be elevated post-operatively, monitor weight and adjust intake as required. (R)

• Aim for energy and protein intakes of at least 30 kcal/kg/day and 1.2 g protein/kg/day in patients receiving radiotherapy or chemoradiotherapy. Patients should have their weight and nutritional intake monitored regularly to determine whether their energy requirements are being met. (R)

• Perform nutritional assessment of cancer patients frequently. (G)

• Initiate nutritional intervention early when deficits are detected. (G)

• Integrate measures to modulate cancer cachexia changes into the nutritional management. (G)

• Start nutritional therapy if undernutrition already exists or if it is anticipated that the patient will be unable to eat for more than 7 days. Enteral nutrition should also be started if an inadequate food intake (60 per cent of estimated energy expenditure) is anticipated for more than 10 days. (R)

• Use standard polymeric feed. (G)

• Consider gastrostomy insertion if long-term tube feeding is necessary (greater than four weeks). (R)

• Monitor nutritional parameters regularly throughout the patient's cancer journey. (G)

• Pre-operative:

○ Patients with severe nutritional risk should receive nutrition support for 10–14 days prior to major surgery even if surgery has to be delayed. (R)

○ Consider carbohydrate loading in patients undergoing head and neck surgery. (R)

• Post-operative:

○ Initiate tube feeding within 24 hours of surgery. (R)

○ Consider early oral feeding after primary laryngectomy. (R)

• Chyle Leak:

○ Confirm chyle leak by analysis of drainage fluid for triglycerides and chylomicrons. (R)

○ Commence nutritional intervention with fat free or medium chain triglyceride nutritional supplements either orally or via a feeding tube. (R)

○ Consider parenteral nutrition in severe cases when drainage volume is consistently high. (G)

• Weekly dietetic intervention is offered for all patients undergoing radiotherapy treatment to prevent weight loss, increase intake and reduce treatments interruptions. (R)

• Offer prophylactic tube feeding as part of locally agreed guidelines, where oral nutrition is inadequate. (R)

• Offer nutritional intervention (dietary counselling and/or supplements) for up to three months after treatment. (R)

• Patients who have completed their rehabilitation and are disease free should be offered healthy eating advice as part of a health and wellbeing clinic. (G)

• Quality of life parameters including nutritional and swallowing, should be measured at diagnosis and at regular intervals post-treatment. (G)

## Introduction

Nutrition and Dietetic services should be organised to provide a seamless service at any stage of the patient pathway. There should be access to dedicated, site-specific dietitians for high-quality service delivery and contribution as a core member of the head and neck multidisciplinary team.^1^ Early identification of high-risk patients and intervention with nutrition support should be included as part of the planning for every patient when treatment options are being considered.^1^^,^^2^ This should include quality of life (QoL) issues to address psychosocial, rehabilitation and survivorship needs of patients and carers.

**Recommendation**

• A specialist dietitian should be part of the multidisciplinary team for treating head and neck cancer patients throughout the continuum of care as frequent dietetic contact has been shown to have enhanced outcomes (R)

## Nutritional screening

The purpose of nutritional screening is to identify patients who are malnourished or at risk of becoming malnourished as early as possible.^1^^,^^2^ All inpatients on admission and all outpatients should be screened to identify those who require early nutritional intervention and prompt referral.^1^^,^^2^ Table I shows the various screening tools available.

**Table I:** Nutritional screening and assessment tools

Screening tool	Information	Validated in cancer patients
The Subjective Global Assessment (SGA) tool	Assesses nutritional status based on features of the history and physical examination	Yes
The patient generated – Subjective Global Assessment (PG-SGA)	An adaptation of the SGA tool for assessing the nutritional status and is patient generated	Yes
The Malnutrition Screening Tool	Compares favourably with the PG-SGA	Yes
The Malnutrition Universal Screening Tool	Currently used by many Trusts across the UK to screen patients	No

## Monitoring

Screening should be repeated weekly for inpatients. For outpatients, weight should be recorded at each outpatient visit and weight loss of 2 kg or more within a two-week period reported to the dietitian.^1^^,^^2^

**Recommendations**

• Patients with head and neck cancer should be nutritionally screened using a validated screening tool at diagnosis and then repeated at intervals through each stage of treatment (R)
• Patients at high risk should be referred to the dietitian for early intervention (R)

## Impact of malnutrition

Patients with head and neck cancer are at risk of malnutrition as a result of the site of their cancer, the disease process and the treatment. Patients may have long standing dietary habits and detrimental lifestyle factors such as alcohol misuse that may predispose them to malnutrition. Regardless of presenting body mass index (BMI), unintentional weight loss of 10 per cent or greater in the preceding six months may lead to a range of problems^3^ as highlighted in Box I.^4^

BOX I **MALNUTRITION ASSOCIATED MORBIDITY**

• Increased risk of infection
• Delayed wound healing
• Impaired function of cardiac and respiratory systems
• Muscle weakness
• Depression
• Poor QoL
• Increased risk of post-operative complications
• Reduced response to chemotherapy and radiotherapy
• Increased mortality rate

Early nutritional intervention is essential to correct pre-existing nutritional deficiencies with regular reviews throughout the patient's journey in order to optimise nutritional status and correct nutrition-related problems at each stage of treatment.^1^^,^^5^

**Recommendation**

• Offer treatment for malnutrition and appropriate nutrition support without delay given the adverse impact on clinical, patient reported and financial outcomes (R)

## Nutritional assessment

Following nutritional screening a full nutritional assessment should be undertaken in a pre-treatment assessment clinic setting and at regular intervals during a patient's treatment trajectory^1^^,^^2^ (Table II).

**Recommendations**

• Use a validated nutrition assessment tool (e.g. scored Patient Generated–Subjective Global Assessment or Subjective Global Assessment) to assess nutritional status (R)
• Offer pre-treatment assessment prior to any treatment as intervention aims to improve, maintain or reduce decline in nutritional status of head and neck cancer patients who have malnutrition or are at risk of malnutrition (G)
• Patients identified as well-nourished at baseline but whose treatment may impact on their future nutritional status should receive dietetic assessment and intervention at any stage of the pathway (G)

**Table II:** Nutritional assessment parameters

Clinical observation	• • Ability to chew and swallow • • Clinical signs of weight loss e.g. ill-fitting dentures/clothing • • Medical history which may affect nutritional intake e.g. coeliac disease, diabetes
Dietary history	Review of recent intake (24 hours recall), with attention being paid to: • • Fluid intake • • Changes in texture • • Reports of fullness • • Length of time and effort taken to eat • • Changes in appetite • • Gastrointestinal function
Calculation of requirements	Energy: • • 25–35 kcal/kg/day dependant on activity level. Can increase further if major complications. Protein: • • 0.8–2.0 g/kg/day for depleted of treatment complications Fluid: • • 30–35 ml/kg/day increases in infection and excessive fluid losses Vitamins and minerals: • • As per recommended daily amounts unless considered deficient
Proposed treatment	• • Disease status, tumour site • • Nutritional implications of previous and current treatment plan
Anthropometry	• • Height • • Weight • • Weight history • • Percentage weight change • • Body mass index; <18.5 kg/m^2^ suggests undernutrition • • Triceps skinfold thickness indicates fat stores • • Mid arm muscle circumference indicates lean tissue mass • • Hand grip strength assesses muscle function
Biochemistry	• • Urea and electrolytes – indicate fluid status although can be disrupted by disease state and treatment • • Albumin – not good indicator of nutritional status due to its long half-life (17–20 days) and it is affected by stress and sepsis • • Pre-albumin – shorter half-life 2–3 days but also affected by infection and stress • • C-reactive protein – indication of acute phase response • • Transferrin – affected by inflammation and infection • • Total lymphocyte count – affected by infection • • Refeeding syndrome risk
Social information	• • Alcohol intake • • Smoking • • Substance misuse • • Social support • • Dentition • • Access to food and cooking skills • • Social and financial circumstances • • Time taken to eat and drink • • Patient perception of nutritional status

## Cancer cachexia

Cachexia syndrome results in decreased appetite, weight loss, metabolic alterations and an inflammatory state that cannot be fully reversed by conventional nutritional support and leads to progressive functional impairment. Pro-inflammatory processes can lead to insulin resistance, increased loss of body fat, muscle mass and production of acute phase proteins. Cytokine-induced metabolic alterations can prevent cachectic patients from regaining body cell mass during nutritional support, and are not relieved by conventional nutritional intervention. Attempts to modulate these changes by other means should be integrated into the management of cancer patients. As a minimal goal body weight should be maintained and further loss prevented. The management approach should be multifactorial and includes assessment and ongoing monitoring with intensive nutritional support, anti-inflammatory treatment, symptom control as well as oncological treatment options to reduce the catabolic effect of the cancer.^6^

## Estimating nutritional requirements

Cancer itself does not have a consistent effect on resting energy expenditure, but may be influenced by oncological treatment. Resting energy expenditure can be unchanged, increased, or decreased.^2^ Cancer patients are mildly hypermetabolic with an excess energy expenditure of between 138 and 289 kcal/day. Total energy expenditure and protein requirements for non-obese ambulatory patients using their actual body weight can be estimated as follows:

Energy, 30–35 kcal/kg/day and protein, 1.2 g/kg/day.^1^ These may be less accurate for severely malnourished, morbidly obese and surgical patients.

**Recommendations**

• Aim for energy intakes of at least 30 kcal/kg/day. As energy requirements may be elevated post-operatively, monitor weight and adjust intake as required (R)
• Aim for energy and protein intakes of at least 30 kcal/kg/day and 1.2 g protein/kg/day in patients receiving radiotherapy or chemoradiotherapy
• Patients should have their weight and nutritional intake monitored regularly to determine whether their energy requirements are being met (R)

## Refeeding syndrome

Refeeding is a syndrome consisting of metabolic disturbances that occur as a result of reintroduction of nutrition to patients who are starved or severely malnourished. It can occur irrespective of the feeding route. The main feature is hypophosphataemia but can feature abnormal sodium and fluid balance; changes in glucose, protein, and fat metabolism, thiamine deficiency, hypokalaemia and hypomagnesaemia.^7^

**Fig. 1:**
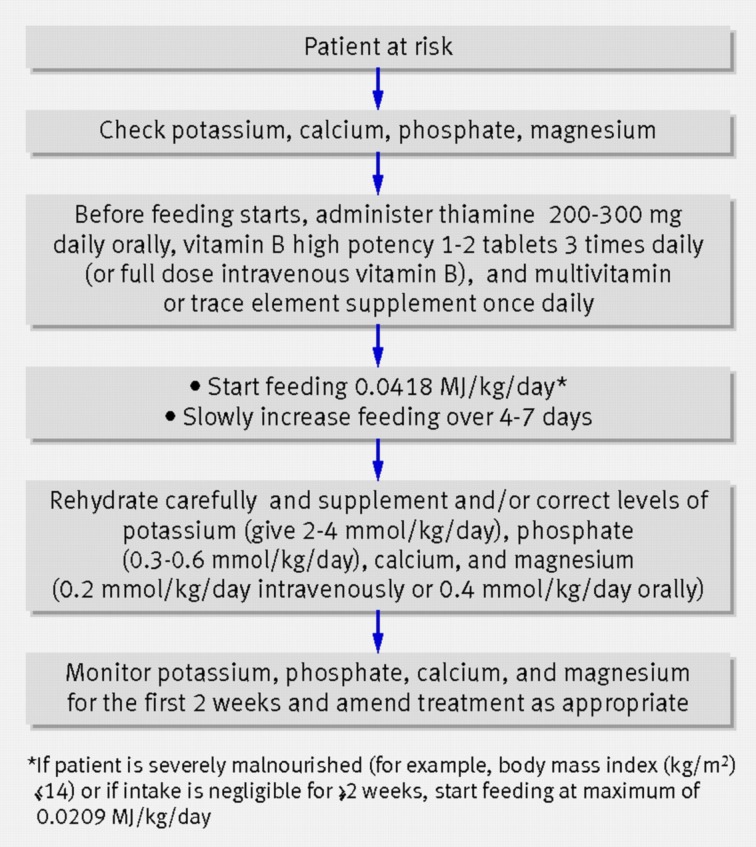
Management of re-feeding syndrome (reproduced with permission from Mehanna *et al*.^7^).

The nationwide incidence of refeeding syndrome in head and neck cancer is unknown. By defining refeeding syndrome as a reduction in serum phosphate to below 0.4 mmol/l,^1^^,^^7^ retrospective data from a regional cancer centre found 37.5 per cent of patients to be at risk as defined by National Institute for Health and Care Excellence criteria (see Box II) with an incidence rate of 9.5 per cent. A suggested management plan for refeeding syndrome is shown in Figure 1.

**Recommendations**

• Perform nutritional assessment of cancer patients frequently (G)
• Initiate nutritional intervention early when deficits are detected (G)
• Integrate measures to modulate cancer cachexia changes into nutritional management (G)

BOX II **CRITERIA FOR DETERMINING PEOPLE AT MODERATE OR HIGH RISK OF DEVELOPING REFEEDING SYNDROME^2^**

Patient has one or more of the following:

• Body mass index less than 16 kg/m^2^
• Unintentional weight loss greater than 15 per cent within last three to six months
• Little or no nutritional intake for more than 10 days
• Low levels of potassium, phosphate, or magnesium prior to feeding

Or patient has two or more of the following:

• Body mass index less than 18.5 kg/m^2^
• Unintentional weight loss greater than 10 per cent within last three to six months
• Little or no nutritional intake for more than 5 days
• A history of alcohol abuse or drugs, including insulin, chemotherapy, antacids or diuretics

## Nutrition support

The aims of nutrition support are to:

• Improve the subjective QoL
• Enhance anti-tumour treatment effects
• Reduce the adverse effects of anti-tumour therapies,
• Prevent and treat undernutrition.

Nutritional support should be considered in the following scenarios:

• Body mass index <18.5 kg/m^2^
• Unintentional weight loss >10 per cent over three to six months
• A BMI <20 kg/m^2^ and unintentional weight loss over three to six months
• Minimal intake >5 days
• Increased nutritional requirements due to catabolism.

### Types of nutrition support

Nutritional intervention should be tailored to meet the needs of the patient and be realistic for the patient to achieve. There are three main methods of nutrition support: oral, enteral and parenteral. Parenteral nutrition support is rarely used in the head and neck setting. It should however be considered if required.

### Oral nutrition support

Nutritional interventions include relaxation of previous therapeutic diets to minimise further nutritional compromise and to positively influence QoL outcomes.^8^ Food fortification is first line advice; however, this may not necessarily be appropriate due to the side effects and intensity of treatment regimens. Patients may require more intensive nutritional support methods from the beginning of treatment over and above traditional food fortification methods with the early use of oral nutrition support, e.g. nutritionally complete liquid supplements. This can be initiated at any point from diagnosis. There are a variety of oral nutritional support products available. The choice will depend on patient preference, current macro and micro nutrient intake and local policy.

### Enteral nutrition (EN) support

The choice of feeding route will depend upon local arrangements, however clinical considerations should include: site of tumour, treatment plan and intent, predicted duration of enteral feeding and patient choice.^9^^,^^10^ The types of tubes available are nasogastric, nasojejunal, tracheo – oesophageal fistulae tubes, orogastric, gastrostomy, gastro-jejunostomy and jejunostomy. Nasogastric, nasojejunal, oro gastric, trachea – oesophageal fistulae tubes are all recommended for short-term use (less than four weeks). National Institute for Health and Care Excellence guidelines on enteral feeding suggest that if enteral feeding is expected to be required for longer than four weeks then gastrostomy insertion is recommended.^2^

Consideration should be made with regard to the timing and method of gastrostomy placement. Screening and assessment for suitability and method of gastrostomy insertion by endoscopic, radiological or surgical approach is essential. Assessment of co-morbidities and contraindications should be undertaken in order to prevent complications of tube insertion prior to oncological treatment. Variation exists for the preferred method of insertion and is dependent on local policy. There are no nationally agreed selection criteria for gastrostomy placement in head and neck patients. Comparison between studies is difficult and made more challenging by limitations in study design as well as the inability to stratify data meaningfully into groups with adequate patient numbers by similar treatment modality, type of gastrostomy and timing of tube placement.^9^^,^^10^ Evidence-based practice guidelines, based on a systematic review of literature across the entire nutrition care pathway, following a National Health and Medical Research Council's process for assessing the level of evidence and evaluating the body of literature, have been published.^1^ Although the optimal method of tube feeding remains unclear,^10^^,^^11^ it is widely accepted that prophylactic tube feeding compared with reactive tube feeding or oral intake alone improves nutritional outcomes with reduced weight loss, and can therefore contribute towards clinical, financial and QoL aspects.^1^^,^^12^ However, high-level evidence base is yet to be generated to confirm the benefits.^13^^,^^14^ Appropriate decision making around prophylactic tube feeding must consider all factors that impact on nutrition including patient demographics, tumour site and staging, impact of treatment modalities on the patient's ability to meet and sustain nutritional requirements, nutritional status, dysphagia, type and placement technique of feeding tube and associated morbidity.^4^^,^^9^^,^^10^ While there is no universally accepted definition of gastrostomy dependency, the principle is recognised and reported.^15^ In clinical studies, gastrostomy tube is used as a proxy measure for poor swallowing in the absence of reviewing nutritional outcome data, intensity and frequency of dietary counselling and swallowing rehabilitation and co-ordination of these services before, during and after treatment.^9^^,^^10^

### Enteral nutrition

The type and volume of EN will depend upon the patients’ symptoms and current intake and is likely to change throughout and following treatment.^2^ There are no data to suggest a role for cancer-specific enteral formulae and standard polymeric feeds should be used in this population group. There are a range of nutritionally complete feeds available. Local policies and feed contract arrangements determine the type and make.

### Immune-enhanced nutrition

Immunonutrition are feeds containing amino acids, nucleotides and lipids. There are no additional benefits to immunonutrition pre-operatively over standard nutrition support. Preliminary data suggest that in the peri-operative period, *N-*3 enriched nutrition support may improve nutritional outcomes including weight, lean body mass and fat mass, reduce post-operative infections and reduce hospital stay.^16^

### Monitoring nutritional support

Monitoring nutritional intervention is essential, as compliance with recommendations can be a problem. Monitoring should involve the multidisciplinary team, including dietitians, medical teams, speech and language therapist and clinical nurse specialists.

**Recommendations**

• Start nutritional therapy if undernutrition already exists or if it is anticipated that the patient will be unable to eat for more than 7 days. Enteral nutrition should also be started if an inadequate food intake (60 per cent of estimated energy expenditure) is anticipated for more than 10 days (R)
• Use standard polymeric feed (G)
• Consider gastrostomy insertion if long-term tube feeding is necessary (greater than four weeks) (R)
• Monitor nutritional parameters regularly throughout the patient's cancer journey (G)

## Nutrition considerations during surgical treatment

Enhanced recovery after surgery programmes are starting to be developed and implemented across Head and Neck Centres. Nutritional interventions are part of enhanced recovery and should be considered at all stages of the pathway from diagnosis to survivorship and wellbeing.

### Pre-operative nutrition

Inadequate oral intake for more than 14 days is associated with a higher mortality. Patients with severe nutritional risk should receive nutrition support for 10–14 days prior to major surgery even if surgery has to be delayed.^5^^,^^16^ Carbohydrate loading is becoming standard practice in some centres for all patients undergoing head and neck cancer surgery. It has been shown to be safe and well tolerated in patients undergoing head and neck surgery. The type of carbohydrate-loading products used will depend on local contractual arrangements. Enteral nutrition is indicated even in patients without obvious undernutrition, if it is anticipated that patients will be unable to eat for more than 7 days peri-operatively. Box III indicates criteria for initiating pre/peri-operative nutrition support and identifies patients with severe nutritional risk.

BOX III **CRITERIA FOR INITIATING PRE-OPERATIVE NUTRITIONAL SUPPORT^2^^,^^5^**

Indications:

• Weight loss >10–15 per cent in 6 months
• Body mass index <18.5 kg/m^2^
• Subjective Global Assessment Grade C
• Serum albumin <30 g/l
• Unable to maintain intake above 60 per cent of recommended intake for more than 10 days

### Post-operative nutrition

Early post-operative tube feeding (within 24 hours) is indicated in patients in whom early oral nutrition cannot be initiated. Nutrition support, especially enteral nutrition, reduces morbidity. In some centres, as part of the enhanced recovery programme, very early nutritional intervention is being trialled. Standard polymeric enteral feeds are suggested post-operatively with currently very limited evidence to support the use of immunonutrition. Early oral feeding after primary total laryngectomy (from as early as 1 day post-operation to 7 days) is thought to reduce length of stay as there has been shown to be no difference in fistulae rates compared with delayed oral feeding of >7 days.

### Nutritional management of chyle leaks

This is a rare complication with an incidence of 1–2 per cent following radical neck dissections, and less common with selective neck dissections often performed in current practice. The management may be conservative, including dietary manipulation or further surgery. A post-operative leak gives the fluid a milky appearance. A triglyceride level >110 mg/dl is diagnostic of a chyle leak. If the triglyceride level is <110 mg/dl, further analysis is required to demonstrate the presence of chylomicrons. A triglyceride level <50 mg/dl usually rules out a diagnosis of a chyle leak unless a patient is malnourished or has been fasted.

The principal aims of nutritional management are to reduce the flow of chyle whilst maintaining nutritional status, ensuring adequate fluid balance and replacing electrolyte losses.

The nutritional management is to use a fat free or high medium chain triglyceride (MCT) product. Medium chain triglyceride is recommended because it is directly absorbed into the portal system resulting in less chyle production. In clinical practice fat free products can be more accessible and practical than MCT feeds. If dietary manipulation is unsuccessful parenteral nutrition may be required. This should not be used as first line management except in extreme cases, e.g. very high-volume leaks (>1000 ml).

There is no consensus on how to nutritionally manage chyle leaks, how long nutrition management should be pursued, or what constitutes an acceptable amount of chyle output.^1^^,^^17^^,^^18^ The nutritional intervention is usually dependant on clinician preference.

**Recommendations**

• Pre-operative:
  ○ Patients with severe nutritional risk should receive nutrition support for 10–14 days prior to major surgery even if surgery has to be delayed (R)
  ○ Consider carbohydrate loading in patients undergoing head and neck surgery (R)
• Post-operative:
  ○ Initiate tube feeding within 24 hours of surgery (R)
  ○ Consider early oral feeding after primary laryngectomy (R)
• Chyle leak:
  ○ Confirm chyle leak by analysis of drainage fluid for triglycerides and chylomicrons (R)
  ○ Commence nutritional intervention with fat free or MCT nutritional supplements either orally or via a feeding tube (R)
  ○ Consider parenteral nutrition in severe cases when drainage volume is consistently high (G)

## Nutritional considerations during curative radiotherapy ± chemotherapy

Concomitant mucositis during radiotherapy ± chemotherapy results in weight loss, which cannot be completely prevented by nutritional counselling alone.^19^ Intensive dietary counselling and oral nutrition support to increase dietary intake and to prevent treatment associated weight loss is recommended for patients undergoing radiotherapy of the head and neck.^20^ This is also advised to prevent interruptions to radiation treatment. Tube feeding is recommended if the cancer interferes with swallowing or if mucositis is anticipated which may interfere with oral and/or pharyngeal swallowing.^21^ The optimal method of tube feeding remains unclear, therefore, the risks and benefits of both proactive and reactive approaches should be discussed by the dietitian with the patient to ensure individualised nutritional care.^1^ Prophylactic tube feeding compared to oral intake alone or reactive tube demonstrates reduced weight loss in the short term, may reduce unplanned hospital admissions and may improve QoL during and after treatment.^1^ The Clinical Oncological Society of Australia recommends that patients should be seen weekly during radiotherapy. However, in some centres twice weekly follow up is provided. Intensity Modulated radiotherapy is now used for the treatment of head and neck cancer. This treatment has not been found to reduce nutrition related toxicity and patients should be managed in the same way as conventional radiotherapy. Patients receiving biological agents such as cetuximab with radiotherapy should be nutritionally managed in the same way as those receiving chemoradiotherapy.^1^

**Recommendations**

• Weekly dietetic intervention is offered for all patients undergoing radiotherapy treatment to prevent weight loss, increase intake and reduce treatments interruptions (R)
• Offer prophylactic tube feeding as part of locally agreed guidelines, where oral nutrition is inadequate (R)

## Nutritional considerations during palliative chemotherapy and radiotherapy

The use of chemotherapy and radiotherapy may be used to relieve symptoms caused by the cancer where the goal is to improve the QoL but not treat the disease. Palliative chemotherapy and radiotherapy is increasingly used in the treatment of head and neck cancer and the dietitian has a role in supporting the nutritional needs of patients receiving these treatments. Patients may experience side effects from these treatments which affect their ability to take adequate nutrition or require dietary intervention to support their QoL.^1^^,^^9^

## Rehabilitation

Patients are at high risk of developing late and long-term effects of treatment resulting in eating difficulties requiring dietary modification, supplementation and alternative feeding. Patients should be seen fortnightly for at least six weeks post-treatment and patients should be reviewed by the dietitian for up to six months or for as long as they require management of chronic toxicities, weight loss or tube feeding.^1^

Guidance for clinical management and a strategic framework for structured head and neck ‘local support’ services as part of the multidisciplinary team are limited, but should be interpreted at a local level to deliver high-quality patient-centred nutritional care.^1^^,^^4^

**Recommendation**

• Offer nutritional intervention (dietary counselling and/or supplements) for up to three months after treatment (R)

## Survivorship

The number of patients living with cancer or its long-term side effects is increasing. Many of our cancer survivor patients have unmet needs. It is recommended that patients are offered education and support events (Health and Wellbeing Clinics) after completion of treatment and rehabilitation.^22^ Dietitians can play a key role in these events by offering tailored healthy eating advice that takes into consideration the long-term side effects that head and neck cancer patients may experience. Macmillan cancer support is currently developing a healthy eating toolkit that can be adapted for use with head and neck cancer patients.

**Recommendation**

• Patients who have completed their rehabilitation and are disease free should be offered healthy eating advice as part of a health and wellbeing clinic (G)

## Quality of life

Head and neck-specific validated tools exist to evaluate QoL. These tools may include factors relating to eating and drinking, but there is no nutrition-specific module to assess the relationship between QoL, nutritional status, malnutrition and nutrition support in this patient group.^4^ Reduction in QoL can be directly related to weight loss and malnutrition with an improvement seen when dietary counselling and aggressive nutritional support is maintained during treatment. The impact of having a feeding tube on patients' QoL requires further evaluation.

**Recommendation**

• Quality of life parameters, including nutrition and swallowing, should be measured at diagnosis and at regular intervals post-treatment (G)

### Key points

• Nutrition has an important role in the management of head and neck cancer and its associated treatment modalities
• Specialist site specific dietitians should be part of the multidisciplinary team for treating head and neck cancer patients as frequent dietetic contact has been shown to enhance outcomes
• Comprehensive nutritional assessment is necessary to ensure early recognition of patients who have or are at risk of developing malnutrition to allow timely and appropriate intervention
• Nutritional interventions are varied and have an important role throughout the course of the disease, from diagnosis through to terminal care
• Effective nutritional interventions should ultimately aim to improve QoL and enhance the beneficial effects of treatment.
